# Functional MRI evaluation of cognitive effects of carotid stenosis revascularization

**DOI:** 10.1002/brb3.2512

**Published:** 2022-03-01

**Authors:** Betty Chinda, Kim H Tran, Sam Doesburg, William Siu, George Medvedev, S Simon Liang, Angela Brooks‐Wilson, Xiaowei Song

**Affiliations:** ^1^ Department of Biomedical Physiology & Kinesiology Simon Fraser University Burnaby British Columbia Canada; ^2^ Clinical Research and Evaluation, Surrey Memorial Hospital Fraser Health Authority Surrey British Columbia Canada; ^3^ Department of Radiology Fraser Health Authority Royal Columbian Hospital New Westminster British Columbia Canada; ^4^ Department of Neurology Fraser Health Authority Royal Columbian Hospital New Westminster British Columbia Canada; ^5^ Department of Medicine University of British Columbia Vancouver British Columbia Canada; ^6^ Canada's Michael Smith Genome Sciences Centre, BC Cancer Vancouver British Columbia Canada

**Keywords:** brain function, carotid stenosis, cognitive function, functional MRI, revascularization

## Abstract

**Introduction:**

Severe internal carotid stenosis, if left untreated, can pose serious risks for ischemic stroke and cognitive impairments. The effects of revascularization on any aspects of cognition, however, are not well understood, as conflicting results are reported, which have mainly been centered on paper‐based cognitive analyses. Here, we summarized and evaluated the publications to date of functional MRI (fMRI) studies that examined the mechanisms of functional brain activation and connectivity as a way to reflect cognitive effects of revascularization on patients with carotid stenosis.

**Methods:**

A PubMed and Google Scholar (covering the relevant literature until November 1, 2021) search yielded eight original studies of the research line, including seven resting‐state and one task‐based fMRI reports.

**Results:**

Findings demonstrated treatment‐related alterations in fMRI signal intensity and symmetry level, regional fMRI activation pattern, and functional brain network connectivity. The functional brain changes were associated largely with improvement in cognitive function assessed using standard cognitive test scores.

**Conclusions:**

These findings support the contribution of fMRI to the understanding of brain functional activation and connectivity changes revealing cognitive effects of revascularization in the management of severe carotid stenosis. The review also highlighted the importance of reproducibility through enhancing experimental designs and cognitive task applications with future research for potential clinical translation.

## Graphical abstract

Controversy exists regarding the cognitive benefits of revascularization for patients with asymptomatic stenosis with the findings based mainly on paper tests. Functional MRI has been used to more sensitively detect changes in brain activation that reflect neurovascular and metabolic mechanisms underlying clinical intervention; however, there lacks a synthesized review of fMRI findings of treatment effects on cognition in patients with carotid stenosis. We thus conducted a PubMed and Google Scholar search to review and analyze the available data and also evaluate the challenges in the research field.

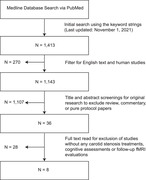

## 1 INTRODUCTION

The narrowing of the internal carotid arteries with plaque buildup in carotid stenosis is a well‐known risk factor for ischemic stroke and death (Flaherty et al., 2013). When the stenosis is ≥70% as per North American Symptomatic Carotid Endarterectomy Trial criteria, it is considered severe and clinical interventions may be recommended even in the absence of overt clinical manifestations (Halliday et al., 2010). Severe stenosis can either be symptomatic where it is characterized by the presence of transient or permanent neurologic or ischemia‐like symptoms or asymptomatic where no obvious neurological dysfunction can be detected on the physical examination by a neurologist and the patient reports no stenosis attributable complaints (Lanzino et al., 2009). Common clinical treatments for severe carotid stenosis include medical therapy and carotid endarterectomy (CEA), for which surgery is performed to remove the plaque. Alternatively, carotid angioplasty and stenting (CAS) is used, in which a mesh‐like stent is inserted into the affected artery to improve blood flow (Lanzino et al., 2009). The choice of treatment is dependent on factors including the severity of stenosis, age, and the presence of comorbidities (Lanzino et al., 2009). While CEA is generally considered as the preferred treatment for symptomatic patients, CAS is typically used for patients who are deemed high risk for invasive surgical procedures such as older and frail patients, those with poor neck structure conditions preventing surgical access to carotid arteries or those with a contralateral artery obstruction (Lanzino et al., 2009). Revascularization treatments for carotid stenosis remarkably reduce the risks of adverse ischemic events (Lanzino et al., 2009; Rothwell et al., 2007).

Depending on the most impacted blood vessels, severe carotid stenosis if not treated can result in significant cognitive impairments, notably in executive functions and working memory (Berman et al., 2007; De Rango et al., 2008). The cognitive impact of stenosis treatment is subject of several reviews, but so far inconclusive findings of revascularization have been reported (Berman et al., 2007; De Rango et al., 2008; Ghogawala et al., 2008; Lal, 2007; Plessers et al., 2014; Sztriha et al., 2009). Berman et al. (2007) found the effects of revascularization inconclusive: with 36% of the studies showing cognitive improvements postrevascularization, whereas the remaining reports seeing mixed (50%) or negative (14%) results. Similarly, Ghogawala et al. (2008) observed posttreatment improvements in 29% of the studies, mainly concerning verbal memory and attention, whereas the other studies reported either a decline (41%) or no change in cognition (29%). In addition, Plessers et al. (2014) identified approximately 10% of patients in several studies showing cognitive improvements post‐CEA, whereas about 10−15% of patients experienced a cognitive decline, highlighting the ambiguity of the cognitive effects of revascularization.

Mixed results shown in these studies may be attributed to several factors, such as cognitive lateralization of neurocognitive effects, heterogeneity of patients’ presentation of symptoms, variability of neuropsychological testing methodologies, statistical analyses, timing of the assessments, and inconsistencies in study design including the lack of control groups (Berman et al., 2007; Ghogawala et al., 2008; Kolb et al., 2019). Most notably, the majority of previous research has solely used paper‐based cognitive tests to examine cognitive impact. Neuroimaging methods on the other hand can more sensitively detect functional brain changes arising from revascularization, while also helping prognostically, to identify patients with the most risks of cognitive decline and dementia (Zhang et al., 2020).

In recent years, functional magnetic resonance imaging (fMRI) has begun to be used in characterizing functional brain changes following revascularization. The fMRI method studies functional brain changes based on its ability to detect brain functional activation utilizing the dynamic paramagnetic properties of capillary blood circulation (perfusion‐weighted imaging) or during oxygen exchange (Blood‐Oxygen‐Level‐Dependent or BOLD imaging) (Ogawa et al., 1990). Detection of brain fMRI activation changes can be coupled with specific fMRI tasks (task‐phase fMRI), where the patterns of fluctuation of the BOLD signals are observed in response to the task. Meanwhile, fMRI activation can also be observed during “task‐free” resting‐state recordings, where there is no implicit cognitive input/output (resting‐state fMRI). With resting‐state fMRI, the changes in the pattern of functional connectivity, that is, the temporal correlation of spontaneous BOLD activations among spatially distributed brain regions at “rest,” can be monitored over time (Smitha et al., 2017). Functional connectivity is usually studied in clusters of neural cells (called networks) responsible for various brain activities. Analyses typically include the salience, default mode, and sensorimotor networks involved in regulating behavior and brain functions, enabling the resting phase and controlling for sensory and motor activities (Smitha et al., 2017).

Although fMRI activation in the brain and cognitive performance are not equal, fMRI can provide a view of how the brain works in response to cognitively demanding tasks, providing a measure that can be used to link with cognitive testing scores and allowing an inference about one's cognitive abilities. For instance, in a well‐designed task‐phase fMRI study, the difference in brain activation changes between task runs and baseline can provide insights into different cognitive states as well as their functional localization in specific brain regions, which can be corroborated when combined with task performance parameters such as accuracy and reaction time. Similarly, resting‐state fMRI studies can be used to characterize normal and abnormal brain functional connectivity in clinical conditions, which can be indicative of cognitive decline (Lv et al., 2018).

FMRI has been widely used to study cognitive changes and treatment effects across disease conditions (Guo et al., 2018). However, there is limited knowledge about using fMRI in accessing cognitive effects of clinical revascularization in treating carotid stenosis. Here, we conduct a review study to identify and map the available fMRI evidence towards understanding the cognitive benefits of carotid stenosis revascularization. To the best of our knowledge, this is the first attempt to summarize fMRI research findings on the cognitive impacts of revascularization in patients with severe carotid stenosis.

## 2 METHODOLOGY

### 2.1 Search terms

We searched the current literature (from January 1, 1990—the initiation of the fMRI technology, until November 1, 2021) using the MEDLINE databases, the National Library of Medicine's premier bibliographic resource. This resource contains 27 million+ references of 5200 journals in life sciences chiefly in biomedicine. We cross‐checked the search results with Google Scholar to ensure adequate inclusion of studies on the topic.

The search was performed by combining the following keyword sets with controlled vocabularies for medical and health fields: Set‐1: “carotid stenosis” or “steno‐occlusive disease” or “carotid occlusion” or “carotid artery stenosis” or “carotid artery disease” or “MH carotid stenosis” or “MH carotid artery diseases.” Set‐2: “revascularization” or “stent” or “endarterectomy” or “stenting” or “CAS” or “CEA” or “carotid angioplasty & stenting” or “carotid endarterectomy” or “carotid stenting” or “carotid intervention” or “MH myocardial revascularization” or “MH cerebral revascularization” or “MH stents” or “MH endarterectomy” or “MH endarterectomy, carotid.” Set‐3: “cognition” or “cognitive” or “memory” or “brain function” or “brain changes” or “brain functional connectivity” or “functional connectivity” or “MH cognition” or “MH memory” or “MH functional status.” Set‐4: “magnetic resonance imaging” or “fMRI” or “MRI” or “functional MRI” or “blood oxygen dependent” or “BOLD fMRI” or “perfusion fMRI” or “arterial spin labeling” or “ASL fMRI” or “MH magnetic resonance imaging” or “MH spin labels.” The initial search yielded 1413 articles.

### 2.2 Inclusion/exclusion criteria

**FIGURE 1:**
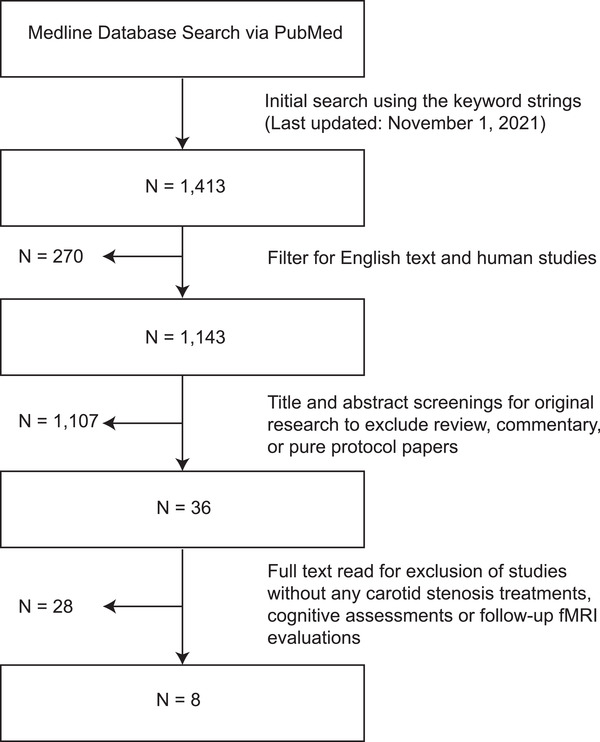
The literature search and selection process

The search was streamlined by including original research on humans in the English language, producing a subset of 1143 articles (shown in Figure 1). The article titles were examined to exclude review, commentary, protocol, and opinion papers. Any studies that did not use fMRI were also excluded, narrowing to 36 papers.

These papers were screened via full‐text analysis to meet the criteria: (1) fMRI studies examining cognitive function in patients with both symptomatic and asymptomatic carotid stenosis, (2) patients received a form of standard clinical revascularization treatment by CAS, CEA or a combination, (3) a baseline cognitive evaluation was performed, and (4) a minimum of one posttreatment cognitive evaluation was performed (shown in Figure 1). A total of eight studies met the criteria and underwent detailed examination with their descriptions presented in the *Results* section below (Figure 1 and Tables 1A and 1B).

**TABLE 1A:** Summary of patient characteristics of the studies under review

First author	Country	Year of publication	Sample size	Age in year (mean ± SD)	Sex male (%)	Degree of ICA stenosis *N* (%)	Stenosis side *N* (%)	Treatment
Cheng	Taiwan	2012	Unilateral asympt CS = 17; HC = 26	71.4 ± 7.3	12 (71%)	≤70 = 6 (35%) ≤80 = 6 (35%) ≤90 = 1 (6%) ≤99 = 4 (24%)	Right = 10 (59%) Left = 7 (41%) Bilateral = 0 (0%)	CAS
Lin	Taiwan	2016	Asympt CS = 25	71.4 ± 7.8	21 (84%)	81.0 ± 9.8 (%)^a^	N/A	CAS
Wang	China	2017	Unilateral asympt CS = 24	66.8 ± 5.8	12 (75%)	≥70 = 16 (100%)	Right = 11 (69%) Left = 5 (31%) Bilateral = 0 (0%)	CAS
Huang	Taiwan	2018	Unilateral asympt CS = 13; HC = 17	69.3 ± 10.7	11 (85%)	78.6 ± 11.3 (%)^a^	Right = 7 (54%) Left = 6 (46%) Bilateral = 0 (0%)	CAS
Tani	Japan	2018	Unilateral sympt CS = 8	69.3 ± 6.2	8 (100%)	≤70 = 4 (50%) ≤80 = 2 (25%) ≤90 = 1 (12.5%) ≤99 = 1 (12.5%)	Right = 2 (25%) Left = 6 (75%) Bilateral = 0 (0%)	CAS
Porcu	Italy	2019	Unilateral asympt CS = 14	73.5 ± 6.2	10 (71%)	N/A	Right = 7 (50%) Left = 7 (50%) Bilateral = 0 (0%)	CEA
Porcu	Italy	2021	Unilateral asympt CS = 20	75.1 ± 6.1	14 (70%)	≥70 = 20 (100%)	Right = 11 (55%) Left = 9 (45%) Bilateral = 0 (0%)	CEA
Chinda	Canada	2021	Unilateral sympt CS = 1 (TIA, aphasia, amaurosis fugax) Unilateral asympt CS = 1	73.0 ± 11.3	2 (100%)	≤70 = 1 (50%) ≤99 = 1 (50%)	Right = 1 (50%) Left = 1 (50%) Bilateral = 0 (0%)	CAS

*Abbreviations*: Asympt CS, asymptomatic stenosis; CAS, carotid angioplasty and stenting; CEA, carotid endarterectomy; characterized by the presence of transient, chronic neurologic or ischemia‐like symptoms; CS, carotid stenosis; HC, healthy controls; ICA, internal carotid artery; lacking ischemia‐like symptoms; M, male; *N*, sample size; N/A, not available; SD, standard deviation; sympt CS, symptomatic stenosis; TE, echo time; TIA, transient ischemic attack.

^a^ Only a range of the degree of ICA stenosis was provided.

**TABLE 1B:** Summary of the fMRI protocols of the studies under review

First author (year)	Experimental design	fMRI condition	fMRI acquisition	Cognitive test(s)	Cognitive/functional domain(s) targeted	Brain ROI investigated	fMRI processing and analysis	Main findings
Cheng (2012)	Two MRI scans pretreatment^a^ and 3 months posttreatment	Resting‐state: eyes opened One session Volume per session = 124	3.0T GE discovery; EPI; TR/TE 3000/30 ms; Flip angle = 90°; FOV = 222 × 222 mm; Voxel size = N/A	MMSE, backward digit span test; immediate and delayed recall test; symbol digit test; Wechsler Adult Intelligence Scale; trail‐making test; Stroop test; modified complex figure test with copy and recall	Working memory, verbal memory, attention, executive function, visuospatial perception	Default mode, frontoparietal and the dorsal attention networks	FSL, smoothing (6‐mm kernel), cluster size (n/a, *p* < .05), ROI voxel‐wise correlation analysis (radius = 4 mm)	Patients had a markedly decreased BOLD functional connectivity between ROIs on the stenotic side, suggesting a disruption of interhemispheric connectivity. Three months post‐CAS, there were small increases in FC between the default mode and frontoparietal networks ipsilateral to the treated ICA. Brain fMRI changes were correlated with improvements in dizziness symptom measure and MMSE score.
Lin (2016)	Two MRI scans pretreatment^a^ and 3 months posttreatment	Resting‐state: eyes opened One session Volume per session = 124	3.0T GE discovery; EPI; TR/TE = 3000/30 ms; Flip angle = 90°; FOV = 222 × 222 mm Voxel size = N/A	Dizziness handicap inventory; MMSE; auditory verbal learning test; modified trail making test; Stroop test; digit modalities test and modified complex figure test	Global cognition, executive function, verbal memory, attention, visuospatial perception	Default mode, dorsal attention, frontoparietal, sensorimotor, salience, and primary visual networks	SPM, smoothing (6‐mm kernel), cluster size (n/a, *p* < .05), ROI voxel‐wise correlation analysis (radius = 4 mm)	Posttreatment patients showed increases in FC strength between regions in the contralateral default mode and dorsal attention network. Patients showed post‐CAS improvement in dizziness alleviation, FC, and neuropsychological scores (MMSE, verbal and visual memory).
Wang (2017)	Two MRI scans 7 days pretreatment and 3 months posttreatment	Resting state (N/A for condition, session #, volume per session)	3.0T Siemens; EPI; TR/TE = 2000/30 ms; Flip angle = 90°; FOV = 240 × 240 mm; Voxel size = 3.75 × 3.75 × 3.8mm^3^	MMSE; MoCA; digit symbol test; Rey auditory verbal learning test; digit span test	Global cognition and verbal memory	Bilateral posterior cingulate cortex	REST; smoothing (8‐mm kernel), cluster size (n/a, *p* < .05), ROI‐based correlation analysis, amplitude of low‐frequency fluctuation analysis	Three months post‐CAS, there was an increase in FC to the posterior cingulate cortex, mainly from the right supra frontal gyrus. Improvements in global cognition and verbal memory (MMSE, verbal memory, and delayed recall tests) post‐CAS were also observed.
Huang (2018)	Three MRI scans 1 week and 1 month pretreatment and 1 year posttreatment	Resting state (N/A for condition or session #) volume per session = 180	3.0T Siemens; EPI; TR/TE = 2000/30 ms; Flip angle = 90°; FOV = 220 × 220 mm; Voxel size = 3.44 × 3.44 × 4mm^3^	MMSE; Raven's standard progressive matrices; Chinese graded word reading test; California verbal learning test‐II; trail making test‐A; Stroop test	Global cognition, episodic memory, executive function, reaction time	Default mode, sensorimotor, salience, dorsal attention, frontal eye field, and frontoparietal networks	REST; smoothing (6‐mm kernel), cluster size (n/a, *p* < .05), seed‐correlation analysis (seed radius = 4 mm)	Pre‐CAS, unilateral CS patients showed decreased FC in the ipsilateral side to stenosis and connectivity in the contralateral hemisphere in the sensorimotor and salience networks. Post‐CAS (at both times), the interhemispheric FC became more symmetrical, mirroring the presentations seen in HCs. Parts of the connections did not return to the HC state even in the 1‐year assessment (e.g., contralateral thalamus‐primary motor cortex hyperconnectivity).
Tani (2018)	Two MRI scans pretreatment^a^ and 6 months posttreatment	Resting state: eyes opened (N/A session #) volume per session = 92	3.0T Toshiba; TR/TE = 4000/25 ms; Flip angle = 90°; FOV = 256 × 256 mm; Voxel size = 2 × 2 × 4mm^3^	Wechsler adult intelligence scale III; Wechsler memory scale–revised	Global cognition, verbal memory, working memory, perception, processing speed, delayed memory, visual memory, attention, and concentration	Default mode network	CONN‐fMRI; smoothing (8‐mm kernel), cluster size (n/a, *p* < .05), independent component analysis	Post‐CAS, FC decreased between the posterior default mode network and the precentral/superior frontal gyrus and left middle frontal gyrus, suggesting an improvement in attention and cognitive control. Verbal intelligence, comprehension, and full‐scale intelligence scores for all patients increased significantly post‐CAS.
Porcu (2019)	Two MRI scans pretreatment^a^ and 3−6 months posttreatment	Resting state: eyes opened (N/A for session #) volume per session = 326	1.5T Philips; EPI; TR/TE = 3000/50 ms; Flip angle = 90°; FOV = N/A; Voxel size = N/A	MMSE	Global cognition	Default mode network	CONN‐fMRIi; smoothing (8‐mm kernel), cluster size (n/a, *p* < .05), seed‐based correlation analysis (seed radius = 5 mm)	Post‐CEA, the medial prefrontal cortex showed increased FC to the right and left cerebellum crus, precuneus, right cerebellum crus, and right middle and superior frontal gyri. An improvement in global cognitive performance (MMSE) post‐CEA was also observed.
Porcu (2021)	Two MRI scans 1 week pretreatment and 12 months posttreatment	Resting‐state: eyes opened (N/A for session #) volume per session = 326	1.5T Philips; EPI; TR/TE = 3000/50 ms; Flip angle = 90°; FOV = N/A; Voxel size = N/A	MMSE	Global cognition	Right precentral gyrus, right middle frontal gyrus, anterior cingulate gyrus	CONN‐fMRI; smoothing (8‐mm kernel), cluster size (n/a, *p* < .05), amplitude of low‐frequency fluctuation analysis	Twelve‐months post‐CEA, there was increased regional neural activity in the right precentral gyrus, middle frontal gyrus, and the anterior division of the cingulate gyrus. MMSE scores saw statistically significant improvements 12 months post‐CEA.
Chinda (2021)	Two MRI scans 1−2 weeks pretreatment and 2 months posttreatment	Task phase: delayed match‐to‐sample two sessions volume per session = 120	3.0T Philips; EPI; TR/TE = 2000/30 ms; Flip angle = 90°; FOV = 240 × 240 mm; Voxel size = 3 × 3 × 3mm^3^	Delayed match to sample task; central nervous system vital signs	Global cognition, working memory, and executive function	Left and right prefrontal cortex, middle temporal lobes	FSL, smoothing (5‐mm kernel), cluster size (*z* > 2.0, *p* < .05). GLM	Post‐CAS, BOLD activations were increased in the treated frontal and temporal lobes in response to the fMRI task. Improvements in accuracy and task completion rates were observed post‐CAS. Decreased fMRI activations in the contralateral hemisphere. Cognitive benefits of CAS were more apparent in the patient with more severe baseline flow limitation due to stenosis.

*Abbreviations*: BOLD, blood‐oxygen‐level‐dependent; CAS, carotid angioplasty and stenting; CEA, carotid endarterectomy; CONN‐fMRI, Matlab‐based software for the computation, display, and analysis of functional connectivity in fMRI; CS, carotid stenosis; EPI, echo planar imaging; FC, functional connectivity; fMRI, functional magnetic resonance imaging; FOV, field of view; FSL, comprehensive library of analysis tools for brain imaging data; GLM, generalized linear model; HC, healthy controls; HI, hemodynamically irrelevant stenosis (not having any symptoms of HR); HR, hemodynamically relevant stenosis (if the ipsilateral middle cerebral artery showed signs of impaired circulation compared to the contralateral middle cerebral artery or if collateral circulation could be detected); ICA, internal carotid artery; MMSE, Mini Mental State Exam; MoCA, Montreal Cognitive Assessment; MRI, magnetic resonance imaging; N/A, not available; REST, resting‐state fMRI data analysis toolkit; ROI, region of interest; SPM, statistical parametric mapping; TE, echo time; temporal correlation of spontaneous BOLD activations among spatially distributed brain regions; TIA, transient ischemic attack; TR, repetition time.

^a^ No specific duration was provided for pretreatment fMRI.

## 3 RESULTS

### 3.1 Overall

Data showed that the earliest fMRI investigation of cognitive effects of the carotid stenosis revascularization was published in 2012 (Cheng et al., 2012). Most studies were conducted using high‐field MRI (3 Tesla), except two that were conducted at 1.5T (Porcu et al., 2019; Porcu et al., 2021). Only one study applied a cognitive task (Chinda et al., 2021), whereas the rest were resting‐state studies (Cheng et al., 2012; Huang et al., 2018; Lin et al., 2016; Porcu et al., 2019; Porcu et al., 2021; Tani et al., 2018; Wang et al., 2017). Clinical revascularization interventions included CAS alone (Cheng et al., 2012; Chinda et al., 2021; Huang et al., 2018; Tani et al., 2018; Wang et al., 2017), CEA alone (Porcu et al., 2019), and combined CAS and medical therapy (Lin et al., 2016). More details of each study are provided below (Tables 1A and 1B).

Sample sizes varied among the studies and ranged from a two‐patient case report to a study of 25 patient participants, with a mean sample size of 15.4 ± 7.9 (median = 18.5). Only two studies included healthy controls (Cheng et al., 2012; Huang et al., 2018). The mean age of patients across the studies was 71.2 ± 7.7 years (median = 71.4), whereas the sex ratio of the research patients was male predominated, ranging from 70 to 100% (mean = 82.0 ± 12.5 %, median = 79.5%). Most of the studies were done on asymptomatic patients (Cheng et al., 2012; Huang et al., 2018; Lin et al., 2016; Porcu et al., 2019; Porcu et al., 2021; Wang et al., 2017); one study investigated only symptomatic patients (Lin et al., 2016); one study investigated both symptomatic and asymptomatic patients (Chinda et al., 2021). Only a couple of the studies (Cheng et al., 2012; Lin et al., 2016) carried out analyses of imaging findings and cognitive assessments blinded to diagnosis and treatment (Table 1A).

Various brain regions and cognitive domains were examined in the studies under review. The task‐phase study involved the administration of a higher‐level working memory task and examined the prefrontal cortex in a small sample (Chinda et al., 2021). Most of the resting‐phase studies examined the default mode network components; although several studies also examined brain regions including the dorsal attention, somatosensory, and salience networks (Table 1B). The Mini Mental State Exam (MMSE) total score, which tests global cognition, was popular among studies where neuropsychological testing was applied (Cheng et al., 2012; Huang et al., 2018; Lin et al., 2016; Porcu et al., 2019; Porcu et al., 2021; Wang et al., 2017); although other tests, such as the Stroop test that measures a range of cognitive domains, were also used by some studies (Table 1B).

### 3.2 Resting‐state fMRI studies

Cheng et al. (2012) compared pre‐CAS and 3 months post‐CAS fMRI data of unilateral asymptomatic carotid stenosis patients with age‐ and education level‐matched healthy controls. The authors observed that post‐CAS, functional connectivity increased between brain regions ipsilateral to the treated internal carotid artery (Smitha et al., 2017). These included areas in the default mode and frontoparietal networks including the hippocampus, cingulate cortex, and medial prefrontal cortex. These brain changes were correlated with improvements in dizziness and MMSE scores. The authors suggested that fMRI showed patterns of brain network disruptions arising from stenosis, and these patterns were consequently improved postintervention (Tables 1A and 1B).

Lin et al. (2016) also examined functional connectivity of carotid stenosis patients pre‐CAS and 3 months post‐CAS with or without medication therapy. The study reported an increase in connectivity strength between the prefrontal and posterior cingulate cortices, in both contralateral default mode and dorsal attention networks. Patients receiving the CAS intervention combined with medication therapies showed greater dizziness alleviation and within‐group improvement in functional connectivity and MMSE verbal and visual memory scores. The study reinforced the importance of combining CAS and medical therapies in treating severe internal carotid artery stenosis (Tables 1A and 1B).

Wang et al. (2017) compared the functional connectivity in asymptomatic unilateral carotid stenosis patients 7 days pre‐CAS and 3 months post‐CAS. The study reported increased connectivity to the posterior cingulate cortex post‐CAS, which correlated with increases in cerebral blood flow to the affected regions and global cognition test scores including MMSE and Montreal Cognitive Assessment, Digit Symbol Test (Chen et al., 2020; Larner, 2018; Nasreddine et al., 2005), and verbal memory and immediate recall tests. The authors concluded that successful CAS resulted in increased brain perfusion and connectivity, and thus, improved cognition (Tables 1A and 1B).

Huang et al. (2018) studied carotid stenosis patients at baseline and two follow‐up time points after CAS, comparing their fMRI activation patterns with those of age‐ and education‐matched healthy controls. Pre‐CAS, patients showed functional hypoconnectivity in the side ipsilateral to stenosis and hyperconnectivity in the contralateral hemisphere in sensorimotor and salience networks (Smitha et al., 2017). One‐month and 1‐year post‐CAS, the interhemispheric functional connectivity gradually became symmetrical, toward the presentations in the healthy controls. The hyperconnectivity of the contralateral thalamus‐primary motor cortex, however, did not return to normal state even at the 1‐year follow‐up. The authors interpreted the hyperconnectivity in carotid stenosis patients as a compensatory mechanism to the neural challenges caused by decreased blood flow, some of which could be changed through CAS (Tables 1A and 1B).

Porcu et al. (2019) evaluated asymptomatic unilateral carotid stenosis patients by contrasting the resting‐phase fMRI data pre‐CEA and 3−6 months post‐CEA. They observed that functional connectivity increased between the default mode network and other regions post‐CEA, which was correlated with improved MMSE test performance (Tables 1A and 1B).

Tani et al. (2018) evaluated unilateral carotid stenosis patients pre‐CAS and 6 months post‐CAS. They reported decreased functional connectivity between the posterior default mode network and the precentral/superior frontal gyrus and left middle frontal gyrus post‐CAS. A CAS‐induced improvement in attention and cognitive control was suggested, whereas verbal intelligence, comprehension, and full‐scale intelligence scores for all patients increased significantly post‐CAS. The study demonstrated the crucial functional connectivity of cortical regions involved in working memory during cognitive recovery (Tables 1A and 1B).

Most recently, Porcu et al. (2021) studied asymptomatic unilateral carotid stenosis patients by contrasting the resting‐phase fMRI data 1 week pre‐CEA and 12 months post‐CEA. They observed treatment‐related increases in regional neural activity in the right precentral gyrus, middle frontal gyrus, and the anterior division of the cingulate gyrus; while the MMSE scores saw statistically significant improvements at the 12 months’ evaluation (Tables 1A and 1B).

### 3.3 Task‐based fMRI studies

Chinda et al. (2021) reported the initial and thus far the only fMRI findings with the use of a cognitive task in two carotid stenosis patients who underwent clinical CAS interventions. The study utilized a delayed match‐to‐sample working memory task with two difficulty levels and cognitive testing using the central nervous system (CNS) vital signs (Gualtieri & Johnson, 2006). Postrevascularization, there was increased fMRI activation in the treated frontal and temporal lobes, which was associated with improvements in accuracy and task completion rates and decreased activation in the contralateral (untreated) hemisphere. The degree of cognitive improvement was related to the degree of flow limitation of the stenosis and the CNS vital signs scores (Tables 1A and 1B).

## 4 DISCUSSION

In this article, we summarized the fMRI findings to date that investigated brain functional changes suggestive of cognitive improvements post clinical interventions for treating carotid stenosis. All studies under evaluation suggested positive treatment effects on fMRI‐based brain functional recovery in patients with severe carotid stenosis after interventions using CAS, CEA, or their combinations (Table 2). The resting‐state studies reported a change in the scale/pattern of the functional connectivity in brain regions including the default mode, sensorimotor, salience, frontoparietal, and visual networks (Cheng et al., 2012; Huang et al., 2018; Lin et al., 2016; Porcu et al., 2019; Porcu et al., 2021; Tani et al., 2018; Wang et al., 2017). Task‐phase fMRI identified increased strength/level of brain activation and increased hemispherical symmetry in the prefrontal cortex in response to higher‐level cognitive stimuli (Chinda et al., 2021). Such effect was seen in the short term (i.e., within 3 months posttreatment) (Cheng et al., 2012; Lin et al., 2016; Porcu et al., 2019; Wang et al., 2017). In the long‐term posttreatment (i.e., 1‐year follow‐up), increased regional neural activity in areas including the precentral gyrus, middle frontal, and the anterior cingulate gyri (Porcu et al., 2021) as well as increased functional connectivity symmetry in the sensorimotor and salience networks (Huang et al., 2018) were also reported. Importantly, such changes in fMRI activation and network connectivity were mirrored by improvements in cognitive performance using paper‐based standard cognitive tests in most studies (Table 2). These findings confirm the recognized sensitivity of fMRI in detecting hemodynamic response, thereby providing a way to view the brain at work in handling challenges, as an effective neuroimaging modality for studying brain function.

**TABLE 2:** Main findings of the fMRI studies to date that investigated cognitive benefits of clinical carotid endarterectomy, carotid angioplasty, and stenting treatments

Main findings	Studies (%)
Functional Connectivity A general increased functional connectivity (FC) to brain regions involved in attention, executive function, and working memory was observed posttreatment; that is, the frontoparietal and the default mode networks and the cingulate cortices.	6 (75)
Cognitive Correlations The increased fMRI connectivity/activation in postcarotid angioplasty and stenting (CAS) and carotid endarterectomy (CEA) patients were correlated with improvements in global cognitive score, for example, MMSE.	7 (87.5)
fMRI Activation Functional activations increased in both CAS and CEA patients, higher amongst those with a less‐severe symptomatic stenosis (≤80%).	4 (50)
Symmetry Blood‐oxygen‐level‐dependent (BOLD) activations became more symmetrical among hemispheres post‐CAS or CEA, indicating functional recovery on the hemisphere ipsilateral to the stenosis.	1 (12.5)

Only one study investigated the influence of symptomatic events such as acute stroke, amaurosis fugax, or transient ischemic attack on the cognitive improvement seen postrevascularization. In the case study using task‐phase fMRI, Chinda et al. (2021) showed the importance of the degree of stenosis prior to the revascularization in determining the prognosis, with a greater post‐CAS increase in BOLD activations in the treated frontal and temporal lobes, in conjunction with improvements in accuracy and task completion rates in an asymptomatic patient with more severe stenosis (>95%) compared with a symptomatic patient with less severe stenosis (only 70%). Previous research has also suggested that symptomatic patients including those with very severe stenosis can experience a greater level of compromise in their cerebral hemodynamics preintervention (Schaaf et al., 2010). How disease history, expression, and symptom severity individually and collectively affect the cognitive recovery following revascularization interventions warrant further research with an increased sample size.

Although all the studies under review suggested posttreatment improvements in brain fMRI activation, it is important to note that considerable heterogeneity exists among these studies in terms of study purpose and design. For example, the domains of cognition examined ranged from higher‐level working memory capacities in the task‐phase studies (Chinda et al., 2021), to visual and verbal memory in the resting phase studies (Table 2). Given the currently thin but growing literature of the research field, all the relevant original fMRI studies that investigated brain functional changes suggestive for cognitive recovery by comparing prerevascularization and postrevascularization have been included in this review paper, regardless of the exact cognitive domain they examined. As a result, a more general statement across all cognitive aspects is unavailable from the studies, although they helped provide data demonstrating the impact of revascularization treatment on cognition, highlighting the advantage of this clinical intervention procedure beyond stroke prevention to better benefit patient care.

The review study also informs future research efforts in this field to investigate brain functional responses with careful selection and implementation of tasks in testing targeted cognitive domains. The well‐needed task‐phase studies can be used to directly view the response of the brain to explicit stimulations when it is at work. fMRI tasks designed on higher‐level functions, such as executive functions and working memory, are appropriate choices in studying cognitive impairment and should be adapted for understanding cognitive recovery in carotid stenosis (Chinda et al., 2021; McDonald et al., 2018). Behavioral data on completing the task such as reaction time and accuracy can also be collected and used in augmenting the fMRI results.

Another source of heterogeneity may revolve around the fMRI processing and analysis techniques utilized (Table 1B). Even though standard fMRI processing and analysis procedures were largely followed by different studies under review, subtle differences in preprocessing such as the size of the smoothing kernel (6 vs. 8 mm) may be related to the subsequent data presentation. Differences were also seen in the cluster size and model of analyses; for example, resting‐state studies used independent component analysis, amplitude of low‐frequency fluctuation analysis, seed‐based correlation analysis, or ROI voxel‐wise analyses. Although each of the methods has advantages (Lv et al., 2018), their differences can make finding generalizations across the studies difficult.

One general criticism of the translational potential of fMRI findings is the dependence of the BOLD signal on efficient capillary gas exchange and respiration and thus can represent an indirect measure of brain activity due to the lag time among other attributing factors (Ogawa et al., 1990). Even so, fMRI offers a unique way to innovatively “see” what happens in the brain when it is at work with sensitivity, relatively high spatial and temporal resolutions, and lack of requirement for iodinated or radioactive substances. The long debate about fMRI, especially BOLD, can only be resolved with its continued development in applications research.

Other than the inherent limitations related to fMRI technology in general, certain limitations with the current studies may be addressed through improving experimental design and methodology. Even though resting‐state fMRI can help understand the general status of functional brain networks through connectivity analysis, task‐based fMRI is required for the identification of the patterns of activation and functional interactions among brain areas, and their alterations, associated with cognitive processes. The fact that there have been notably fewer studies employing fMRI tasks may reflect the challenging nature of task‐based fMRI research from proper design to execution, in contrast to simpler resting‐state fMRI research, where participants lie relaxed and exert minimal cognitive effort in the scanner.

In addition, the period posttreatment that the effect of intervention should be tested is critical for fMRI result interpretation. Most studies scanned patients after a minimum of 1‐month postintervention to focus on sustainable functional change in the brain (Cheng et al., 2012; Chinda et al., 2021; Huang et al., 2018; Lin et al., 2016; Porcu et al., 2019; Tani et al., 2018; Wang et al., 2017). This may present a strategy used to mitigate participant dropout, which is common in longitudinal studies. However, given the high likelihood of a progressive course of functional recovery during a year post‐CAS (Huang et al., 2018), having a baseline and more follow‐up fMRI sessions during the first 6–12 months would allow a better understanding of the short‐ and medium‐term cognitive benefits of revascularization and other interventions.

Including a comparison metric of a matched healthy control group can greatly augment the fMRI findings (Chinda et al., 2021; Lin et al., 2016; Porcu et al., 2019; Tani et al., 2018; Wang et al., 2017). By comparing data between patients and matched controls, a baseline standard is set, against which the level of recovery can be assessed. Similarly, follow‐up data of control participants can inform the general longitudinal change in a cohort to help understand and control the possible influence of changes in age and other environmental and epidemiological aspects, temporal circulation, and other physiological responses to acute interventions, on fMRI data. This recommendation applies to further research, although it can require increased work and budget.

The interpretation of the fMRI results under review deserves some caution. Since BOLD fMRI depends on efficient gas exchange, the signal might be even impacted particularly in patients with widespread cerebrovascular disease such as carotid stenosis. Also, patients operated for symptomatic carotid stenosis may have experienced a cerebrovascular event that acutely impacted cognition and brain perfusion. Cognitive recovery in such participants may be experienced independently of treatment. Even so, many of the studies investigated asymptomatic patients (Cheng et al., 2012; Chinda et al., 2021; Huang et al., 2018; Lin et al., 2016; Porcu et al., 2019; Wang et al., 2017) and the improvements in cognition observed are likely reflecting the actual treatment effect as opposed to natural recovery following an ischemic event.

Our review study has some limitations. First, the search was MEDLINE databases based, Google Scholar supplemented, and targeted publications in English. Although the coverage was wide and high‐quality journals were included, work reported in non‐peer‐reviewed publications, conference papers, and in other languages was excluded. A few recent fMRI studies were excluded as they did not include a treatment (Chang et al., 2016; Goode et al., 2009; He et al., 2020; Liu et al., 2020; Porcu et al., 2020; Xiao et al., 2018), conduct both precognitive and postcognitive tests (Liu et al., 2020; Muscas et al., 2019; Xiao et al., 2018), or examine the internal carotid arteries (Rosen et al., 2018). Similarly, a couple of task‐phase fMRI studies that examined only motor recovery using a motor task without additional pre/postrevascularization cognitive evaluation were excluded (Jensen et al., 2008; Schaaf et al., 2010). Given the relatively small number of available data, the review study is not meant to be a meta‐analysis. The selection of studies was based on the fulfillment of the inclusion criteria and not necessarily the quality of the original work, the specific revascularization procedures, or fMRI experimental and analysis methods. Variability existed in the reviewed studies in terms of sample size, participant condition, follow‐up duration, and rate, impeding the generalizability of findings. The review also did not intend to target specific cognitive domains or brain regions involved and encompassed diverse measures.

Even with these limitations, this review can make a useful contribution to the literature. To the best of our knowledge, this is the first effort to identify, categorize, and summarize research on the cognitive impacts of revascularization using fMRI in patients with severe carotid stenosis. The review, albeit on a relatively small number of available studies, showed that cognitive benefits beyond stroke prevention may be actualized through revascularization. The review identified the need for more specific fMRI studies, especially those that employ proper designs, that is, applying appropriate cognitive tasks, multiple follow‐ups, control conditions, and behavioral and standard cognitive measures.

## 5 CONCLUSION

fMRI is a unique way to provide valuable brain activation information to investigate mechanisms of cognitive effects of clinical revascularization for the treatment of severe carotid stenosis. Recent fMRI research has suggested positive cognitive effects of revascularization in treating severe carotid stenosis, though with notable heterogeneity. The literature review helps facilitate future development of the research field for potential translation.

## CONFLICT OF INTEREST

The authors have no conflicts of interest to declare.

## AUTHOR CONTRIBUTIONS

B. C. conducted the literature review and evaluation, prepared and summarized the results, and drafted the initial version of the manuscript. K. H. T. helped with the literature search and evaluation, results summary, and manuscript editing. W. S. and G. M. enabled the funding support, provided medical consultations, and reviewed the manuscript. S. D. and A. B. W. helped in student supervision, results interpretation, and reviewing the manuscript. S. L. helped in the evaluation and manuscript editing. X. S. enabled funding support, conceptualized and supervised the study, and codrafted the manuscript. All authors participated in revisions and agreed upon publication of the final version of the paper.

### PEER REVIEW

The peer review history for this article is available at https://publons.com/publon/10.1002/brb3.2512

## Data Availability

Data sharing is not applicable to this review article as no new data were generated or analyzed in this study.
